# Mind your step: learning to walk in complex environments

**DOI:** 10.1007/s00221-020-05821-y

**Published:** 2020-05-13

**Authors:** Rachel Mowbray, Dorothy Cowie

**Affiliations:** Department of Psychology, University of Durham, Durham, DH1 3LE UK

**Keywords:** Walking, Development, Planning, Obstacles

## Abstract

In everyday contexts, children must respond to both self-related constraints (their own skills and abilities) and environmental constraints (external obstacles and goals). How do young children simultaneously accommodate these to support skilled and flexible behaviour? We used walking in a complex environment as a testbed for two hypotheses. Hypothesis 1: children will accommodate the self-related constraint of high foot placement variability via dynamic scaling. Hypothesis 2: children will plan ahead, even in complex environments. In our task, 3- to 5-year-olds and adults walked over obstacle sequences of varying complexity. We measured foot placement around the first obstacle in the sequence. Hypothesis 1 was partially supported. In simple, single obstacle environments, children engaged in dynamic scaling like adults. Those with more variable foot placement left greater margins of error between the feet and the obstacle. However, in complex, multiple obstacle settings, children employed large, un-tailored margins of error. This parallels other multisensory tasks in which children do not rely on the relative variability of sensory inputs. Hypothesis 2 was supported. Like adults, children planned ahead for environmental constraints. Children adjusted foot placement around the first obstacle depending on the upcoming obstacle sequence. In doing so, they demonstrate surprisingly sophisticated planning. We, therefore, show that in the motor domain, even very young children simultaneously control both self-related and environmental constraints. This allows flexible, safe and efficient behaviour.

**Electronic supplementary material:**

The online version of this article (10.1007/s00221-020-05821-y) contains supplementary material, which is available to authorized users.

## Introduction

Motor development is an excellent testbed for studying development more generally (Adolph et al. 2018). Motor tasks can be naturally complex, with multiple, overlapping action sequences. Further, motor responses are directly observable—unlike cognitions, which are far more elusive. In this paper, we use walking to explore how children accommodate both environmental constraints (external stimuli) and self-related constraints (skills/abilities) to produce skilled and flexible behaviour in complex environments. To safely walk across a cluttered playroom requires planning ahead for upcoming environmental constraints like toys, furniture and playmates. Foot placement must be adjusted to avoid tripping, falling, or colliding with upcoming obstacles (Chen et al. 1994; Krell and Patla 2002; Matthis et al. 2017; Patla amd Vickers 1997). Adults manage this complex task with relative ease. For a child, the challenge is increased by the significant, self-related constraints of immature balance (Woollacott and Shumway-Cook 1990) and highly variable walking (Hausdorff et al. 1999; Snapp-Childs and Bingham 2009). Using motion capture, we examine the ways in which children accommodate both self-related and environmental constraints in a complex environment. This provides a rare opportunity to directly quantify children’s complex planning abilities.

Infants engage in motor planning. 6-month-olds reach with one or two hands based on the size of the target object (Clifton et al. 1991); at 14 months, infants reach more slowly for objects which must be subsequently placed more carefully (Gottwald et al. 2017). Prospective control is even apparent in the very first movement unit of the reach (Gottwald and Gredebäck 2015). This adaptive motor planning is also apparent in early walking, where infants use vision to detect salient constraints in the environment, and modify their locomotor choices accordingly. Infants use vision to detect salient constraints in the environment, and modify their locomotor choices based on this information. Infants opt, for example, to safely slide down risky slopes, rather than walk (Adolph et al. 1993); or to use handrails for steady crossing of narrow bridges (Berger and Adolph 2003). By doing so, they also accommodate the self-related constraints of the infant motor system—notably, relatively weak muscles and poor balance (Adolph et al. 2003). However, this is only possible after significant experience (Schmuckler 1996). Further, in these experimental examples, there is a single, salient obstacle or barrier to be confronted. By contrast, infants begin waking in environments which are changeable and cluttered with obstacles. In these complex environments, novice walkers fall on average 17 times per hour (Adolph et al. 2012). Further, despite the capacity to visually fixate distal objects (Franchak et al. 2011), infants often walk around their environment in an exploratory manner, without directing locomotion towards clearly discernable goals or locations (Cole et al. 2016). Therefore, for very young walkers, detecting and accommodating both self-related and complex environmental constraints may be challenging.

By 7 years, self-related constraints are much reduced but have not disappeared entirely. Despite years of rich walking experience, balance and movement variability are still not adult-like (Godoi and Barela 2008; Hausdorff et al. 1999). Nonetheless, 7-year-olds do modify their walking to cope with persistent self-related constraints and complex environments. To avoid collisions, they reduce walking speed when approaching obstacles, particularly in low-light environments (Berard and Vallis 2006). They maintain balance by adopting a wider, more stable stance in preparation for the second obstacle of a series (Berard and Vallis 2006). Further, in these multiple obstacle environments, 7-year-olds maintain greater distance between the toe and the obstacle than adults (Berard and Vallis 2006). This reduces the risk of tripping. Therefore, we see significant improvements in the ability to accommodate both environmental and self-related constraints between infancy and 7 years. But how early do these developments come about? Do children younger than 7 show similar adaptive behaviour?

In this paper, we examine the intervening age of 3- to 5-years. Are children this young able to make efficient, adaptive plans in a complex environment? Self-related constraints are very apparent at 3- to 5-years. Although simple walking has the pendulum-like mechanical characteristics of adult gait (Hausdorff et al. 1999; Ivanenko et al. 2004; Okamoto et al. 2003), balance is still poor (Godoi and Barela 2008; Woollacott and Shumway-Cook 1990) and movement variability high (Hausdorff et al. 1999; Snapp-Childs and Bingham 2009). Further, the way in which 3- to 5-year-olds might deal with planning in a complex environment is questionable. Some research shows sophisticated prospective motor control in infants—for example on reaching tasks (Clifton et al. 1991; Gottwald et al. 2017; Gottwald and Gredebäck 2015). However, other research suggests that preschoolers’ planning skills are relatively poor on other motor (Adalbjornsson et al. 2008; Rosenbaum et al. 2013) and non-motor tasks (Zelazo and Carter 1997). We expand on these points below.

Children of 3- to 5-years must contend with significant self-related constraints. In particular, compared with 6- to 7-year-olds, 4-year-olds show high movement variability even in a simple, single obstacle environments (Snapp-Childs and Bingham, 2009). They also have poor static and dynamic balance (Woollacott and Shumway-Cook 1990). However, in simple, single obstacle-crossing tasks, young children accommodate their variable movement to mitigate the risk of trips and falls. Children as young as 4 years engage in Dynamic Scaling (Snapp-Childs and Bingham 2009). That is, those with more variable movement leave larger margins of error between their feet and an obstacle. These margins of error are carefully scaled to the child’s individual level of movement variability (Snapp-Childs and Bingham 2009). Therefore, our first hypothesis (Hypothesis 1) is that children of 3- to 5-years will accommodate the self-related constraint of high movement variability. We predict that they will do this via Dynamic Scaling. We expect this even in complex, multiple obstacle environments because it facilities safe and efficient walking. However, we note that in other complex multisensory tasks, children under 8 years do not *consistently* rely on the relative variability of sensory inputs (Gori et al. 2008; Nardini et al. 2008, 2013). This contrasting result highlights the need for empirical data on how young children respond to self-related constraints in complex behavioural tasks.

Children must also adapt to complex environmental constraints. This can be done by planning ahead—for example, prospectively adjusting foot placement in response to upcoming environmental constraints (Krell and Patla 2002; Matthis et al. 2017). This avoids the need for last-minute, emergency adjustments which can threaten balance (Von Hofsten 2007). At 7 years, children plan for obstacle encounters and reduce walking speed in challenging environments (Berard and Vallis 2006). For younger children, existing data are sparse, but suggests that children of 3- to 5-years may plan ahead. In cluttered environments, the limited data we have suggest that children as young as 4 years fixate obstacles a few steps in advance (Franchak and Adolph 2010). This is similar to adult behaviour (Franchak and Adolph 2010; Matthis et al. 2018; Patla and Vickers 2003). Therefore, our second hypothesis (Hypothesis 2) is that 3- to 5-year-old children will plan ahead in complex environments. We predict that children will adjust foot placement and reduce walking speed in response to upcoming environmental constraints (Berard and Vallis 2006). We would expect this since even infants show sophisticated prospective control of reaching movements—even in complex or uncertain environments (Clifton et al. 1991; Gottwald et al. 2017; Gottwald and Gredebäck 2015). However, we note that young children are notably poor at other types of planning. This includes manual motor tasks such as end-state comfort (Adalbjornsson et al. 2008; Rosenbaum et al. 2013); as well as cognitive tasks (Zelazo and Carter 1997). The present empirical investigation will indicate whether young children are able to plan ahead in the context of multiple environmental constraints.

In this study, 3- to 5-year-old children walked through a complex environment with obstacles. We propose two hypotheses. Hypothesis 1: Children will accommodate the self-related constraint of high foot placement variability. Our specific prediction is that they will do this via Dynamic Scaling (Snapp-Childs and Bingham 2009). This study is the first (to our knowledge) to test this in complex, multiple obstacle environments. Hypothesis 2: Children will plan ahead, even in complex environments. Here, we manipulated the complexity of the environment by manipulating the complexity of an obstacle series in the walking path. We measured foot placement around the first obstacle of the series, since this is crucial for maintaining stability and avoiding collisions or falls. We specifically predicted that children would plan ahead by reducing walking speed and adjusting foot placement around the first obstacle depending on changes in the upcoming obstacle sequence (Berard and Vallis 2006; Krell and Patla 2002; Matthis et al. 2017). In the absence of sufficient existing data, we did not make specific predictions about whether children would plan ahead to a greater or lesser extent than adults. In summary, we expected that children would be sensitive and responsive to both the constraints of the self (variability) and the environment (obstacles). We expected children to use information about the self and the environment to make adaptive motor decisions, which we measured in detail using motion capture. This rich data allow for the sophisticated analysis of children’s ability to plan for environmental complexity and to accommodate self-related constraints.

## Methods

### Design

**Fig. 1:**
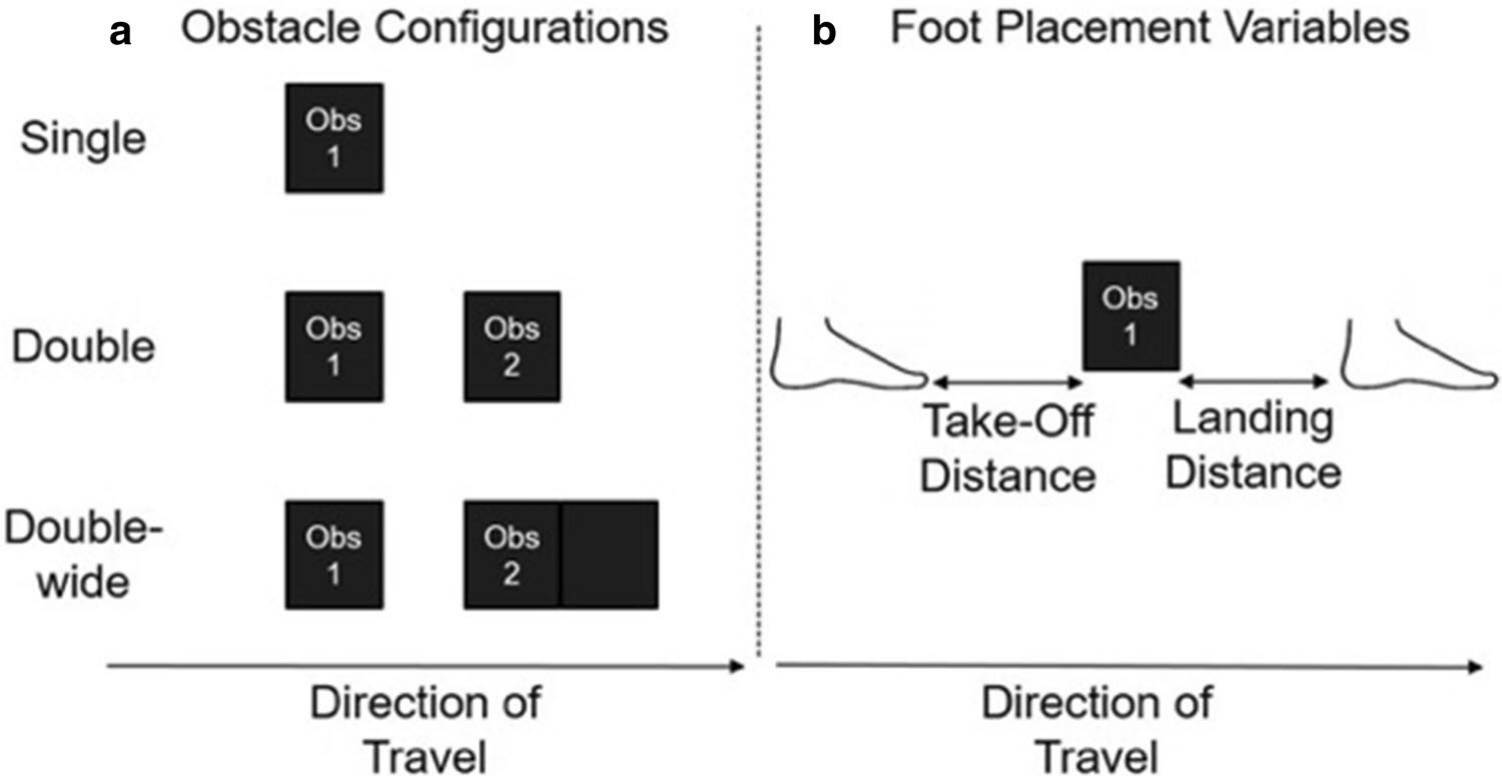
**a** Obstacles were three dimensional boxes. In the single condition, only one obstacle was presented. In the double and double-wide conditions, two obstacles were presented. In the double-wide condition, the second obstacle was twice as wide (front to back) as a standard obstacle. **b** We recorded relevant distances between the feet and the first obstacle of a series as shown above.

There were two independent variables: age (between-subjects: adults and 3- to 5-year-olds) and condition (within-subjects: 3 obstacle conditions: single, double and double-wide, Fig. 1a). We recorded with motion capture the foot placement around the first obstacle of the series, both before the participant crossed the obstacle (take-off) and after they had crossed the obstacle (landing; Fig. 1b). For take-off, we calculated the mean distance between the toes and the front edge of the obstacle. For landing, we calculated the mean distance between the heel and the back edge of the obstacle. We report these mean distances separately for the first foot to cross the obstacle (leading) and the second foot to cross the obstacle (trailing). We also calculated intra-individual variability (standard deviation) for these distances. Thus, in total we have 8 dependent variables (leading/trailing foot x take-off/landing placement × placement mean/variability). For ease of comparisons across ages, data are reported as a percentage of leg length (LL%). A measurement of approach speed (as a percentage of leg length per second) was used as a covariate in supplementary analyses (Supplementary Material). Walking speed for a given trial was calculated between two points prior to obstacle-crossing: (i) two footfalls prior to obstacle crossing and (ii) placement of the trailing foot before the obstacle.

### Participants

Participants had no reported motor or coordination deficits, or developmental disorders. Fifteen 3- to 5-year-olds (10 female) with a mean age of 4.8 years (SD = 0.7 years, range = 3.8–5.7 years), mean leg length of 53.6 cm (SD = 5.3 cm) and mean height of 109.2 cm (SD = 5.6 cm) were recruited via the Durham Developmental Group Families Database. Fifteen adults (11 female) with mean age of 22.6 years (SD = 2.6 years, Range = 18.3–28.3 years), mean leg length of 89.9 cm (SD = 1.5 cm) and mean height of 165.9 cm (SD = 6.8 cm) were recruited via opportunity sampling. The study was approved by the Psychology Department Ethics Committee and carried out according to the principles laid down in the 1964 Declaration of Helsinki.

## Equipment and materials

We used a 16-camera Vicon Nexus motion capture system (240 Hz) under constant lighting levels. Four reflective markers were attached to three-dimensional, box-shaped obstacles and to participants’ bare feet (marker locations on each foot: second metatarsal head, fifth metatarsal head, lateral malleolus and heel). Obstacle size and configuration were scaled to participant leg length. Previous obstacle-crossing studies with older participants did not scale obstacles or configuration to participant size (Krell and Patla 2002; Berard and Vallis 2006). Since leg length positively correlates with step length (Sutherland, 1997), children, with shorter legs, faced a more challenging task than adults. Additionally, the taller the individual, the larger the area of the ground which cannot be seen from a neutral head position (Franchak and Adolph 2010). Therefore, in previous work (Berard and Vallis 2006) children may have had greater opportunity to visually sample obstacles simply due to their shorter stature.

Participants were sorted into bands according to leg length (4 bands: ≤ 49 cm, ≥ 50 and  ≤ 69 cm, ≥ 70 and ≤ 89 cm, and ≥ 90 cm). Obstacle size and configuration were then chosen according to the average leg length value for that band. Obstacle height for each band was 25% of the average leg length value for that band (25 LL%). This height was selected since people preferentially circumnavigate larger obstacles (> 50% LL high) as opposed to stepping over them (Patla et al. 1991), which was not the focus of the present study. All obstacles were a standard 25% LL wide (i.e. front to back) in the single and double conditions. In the double-wide condition, the first obstacle was 25% leg length wide, whilst the second obstacle was 50% LL wide (Fig. 1a). The distance between the start position and the first obstacle was 300% LL. Distance between the two obstacles was 100% LL. A finish line was marked at a distance of 150% LL from the back of the wider second obstacle. Obstacles were presented on a flat, carpeted walkway in linear series. As an example of the scaling procedure: a child with leg length 55 cm would fall into the second band. In this band, the average leg length is 60 cm. For this band, obstacle height would be 15 cm (25% LL) and obstacle width (front to back) would be 15 cm (25% LL). Pre-obstacle distance would be 180 cm (300% LL), inter-obstacle distance would be 60 cm (100% LL) and post-obstacle distance would be 90 cm (150% LL).

### Procedures

Participants wore comfortable clothing (e.g. t-shirt and shorts) and completed the task barefoot. Each participant’s leg length (cm) was measured from anterior superior iliac spine (pelvis) to medial malleolus (ankle) via the medial knee, and markers were attached to the feet. The participant waited behind the starting line until asked to begin each trial. During the double and double-wide conditions, participants were asked how many obstacles they could see before they started walking. This was to clarify that they had perceived both obstacles. Participants were asked to walk in a normal manner and at their usual pace, forwards and across the obstacles. At the finish line, they chose a puzzle piece from a box and carried it back to a simple puzzle on a table near the start line (taking a route to the side of the obstacles). They completed the puzzle with help from an adult. The puzzle game served to motivate children and encouraged natural gait by giving children a fun game to lessen the focus on the walking element of the study.

Piloting indicated that when crossing obstacles people adopt one of two walking patterns. In *Pattern A*, the leading foot crosses the first obstacle; then the trailing foot crosses the first obstacle; then the leading foot crosses the second obstacle. In *Pattern B*, the leading foot crosses the first obstacle; then the trailing foot crosses the first and second obstacles together. To ensure valid comparisons across trials and participants, participants were encouraged to adopt Pattern A. If a participant did not walk as instructed, they were advised to adopt the appropriate walking pattern and the trial was re-run at the end. Participants completed 5 trials per condition, with conditions presented in a random order, totalling 15 trials.

### Data analysis

Motion capture data were filtered using a 6 Hz low-pass Butterworth filter and exported. A custom MATLAB script allowed manual selection of the relevant footfalls and automated calculation of dependent variables. Some trials were excluded from the analysis due to technical problems, experimenter error, or the wrong step pattern. In sum, four adults had one trial excluded, five children had one trial excluded, and one child had two trials excluded. Results were analysed via mixed model ANOVA with factors age (3- to 5-year-olds and adults) and obstacle condition (single, double, double-wide). We used Levene’s test of equality of variance to determine the suitability of the data for ANOVA. There was equality of variance (*p*’s > 0.05) for all data except landing variability for the leading foot in the double-wide condition (*p* = 0.043) and approach speed in the double (*p* = 0.024) and the double-wide (*p* = 0.006) conditions. Nonetheless, given our equal group sample sizes, we consider ANOVA a robust analysis for our data (Field 2013). Main effects were followed up using Bonferroni-corrected post hoc tests and a Greenhouse Geisser correction was applied when the sphericity assumption was not met. Significant interactions were followed up using repeated measures ANOVA. Pearson correlations were used to explore relationships between the mean and variability of foot placement. As a supplement (Supplementary Material), approach speed was added to the analysis as a covariate.

## Results

We present results for foot placement before the first obstacle (take-off), then results for foot placement after the first obstacle (landing). Our results address two hypotheses.

### Hypothesis 1

Children will accommodate the self-related constraint of high foot placement variability.

Specifically, we predicted main effects of age on foot placement, such that children would place their feet further from the obstacle and more variably than adults. We also predicted that those with higher foot placement variability would place their feet further from the obstacle (i.e. Dynamic Scaling; Snapp-Childs and Bingham 2009).

### Hypothesis 2

Children will plan ahead, even in complex environments.

Specifically, we predicted main effects of condition, such that children and adults would change foot placement in response to changes in obstacle condition. We also expected children and adults to reduce walking speed when approaching obstacles. We did not have specific predictions regarding children’s planning abilities relative to adults. Therefore, we did not make specific predictions about interactions between age and obstacle condition.

## Before the first obstacle (take-off)

### Leading foot

Hypothesis 1 was not supported. Neither mean take-off distance nor intra-individual variability for the leading foot was affected by age (*p’s* > 0.2). Hypothesis 2 was partially supported. While mean take-off distance for the leading foot was consistent across obstacle conditions (*p* = 0.19), variability of leading foot placement (Fig. 3a) changed significantly, *F*(2, 56) = 4.1, *p* = 0.021, *ηp*^2^ = 0.129. At both ages, the leading foot was placed more consistently at take-off in the double condition (*M* = 12.9% LL) than in the single condition (*M* = 17.5% LL), *p* = 0.023, with other differences not significant (*p’s* > 0.2). There was no significant interaction between age and condition on mean leading foot placement or intra-individual variability (*p’s* > 0.05).

### Trailing foot

**Fig. 2:**
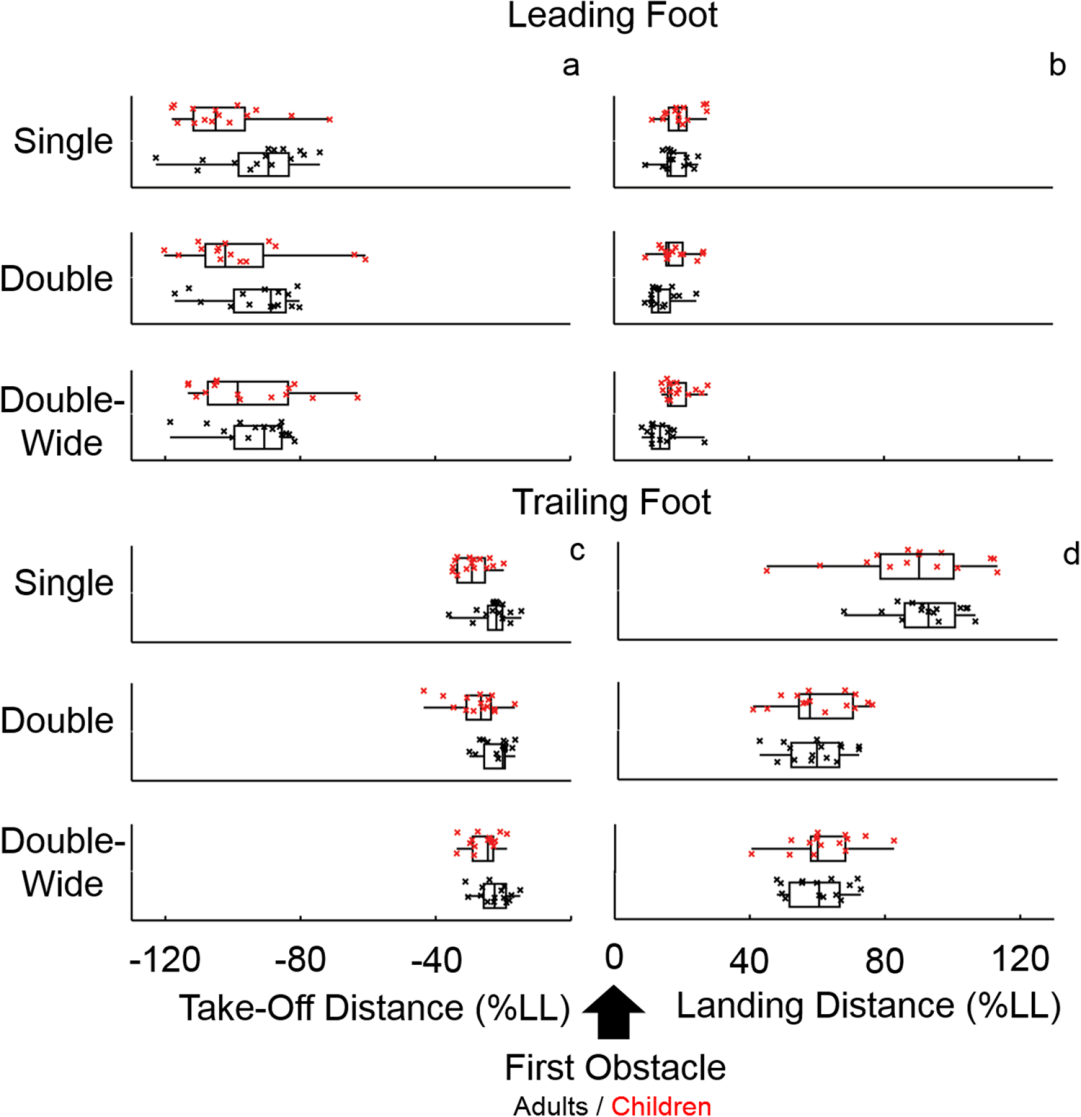
Foot placement around the first obstacle for adults (black) and children (red). The direction of travel is represented from left to right, and zero signifies the location of the first obstacle. Distance is given as a percentage of leg length (LL%) and is shown for each obstacle condition (single, double and double-wide), both before the obstacle (Take-Off Distance—**a**–**c**) and after the obstacle (Landing Distance—**b**–**d**), for both the leading and trailing foot. Each box plot shows the median, lower quartile and upper quartile of the data, as well as individual points

**Fig. 3:**
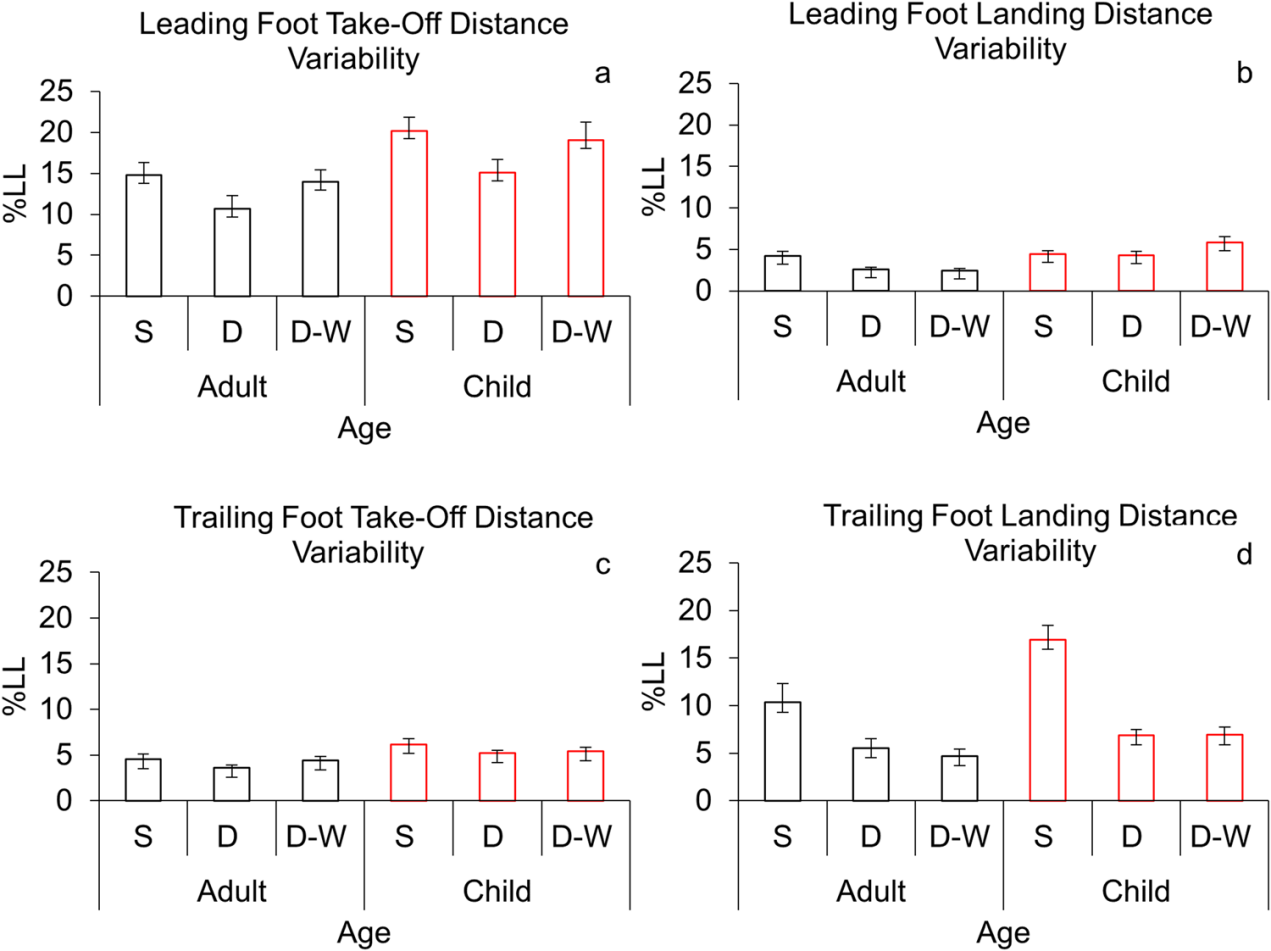
Intra-individual variability (standard deviation) of foot placement around the first obstacle, presented as a percentage of leg length (LL%). Means are given for each condition (*S* single, *D* double, *D-W* double-wide), age group (adults: black; children: red), and foot (leading and trailing). Error bars represent standard errors

**Fig. 4:**
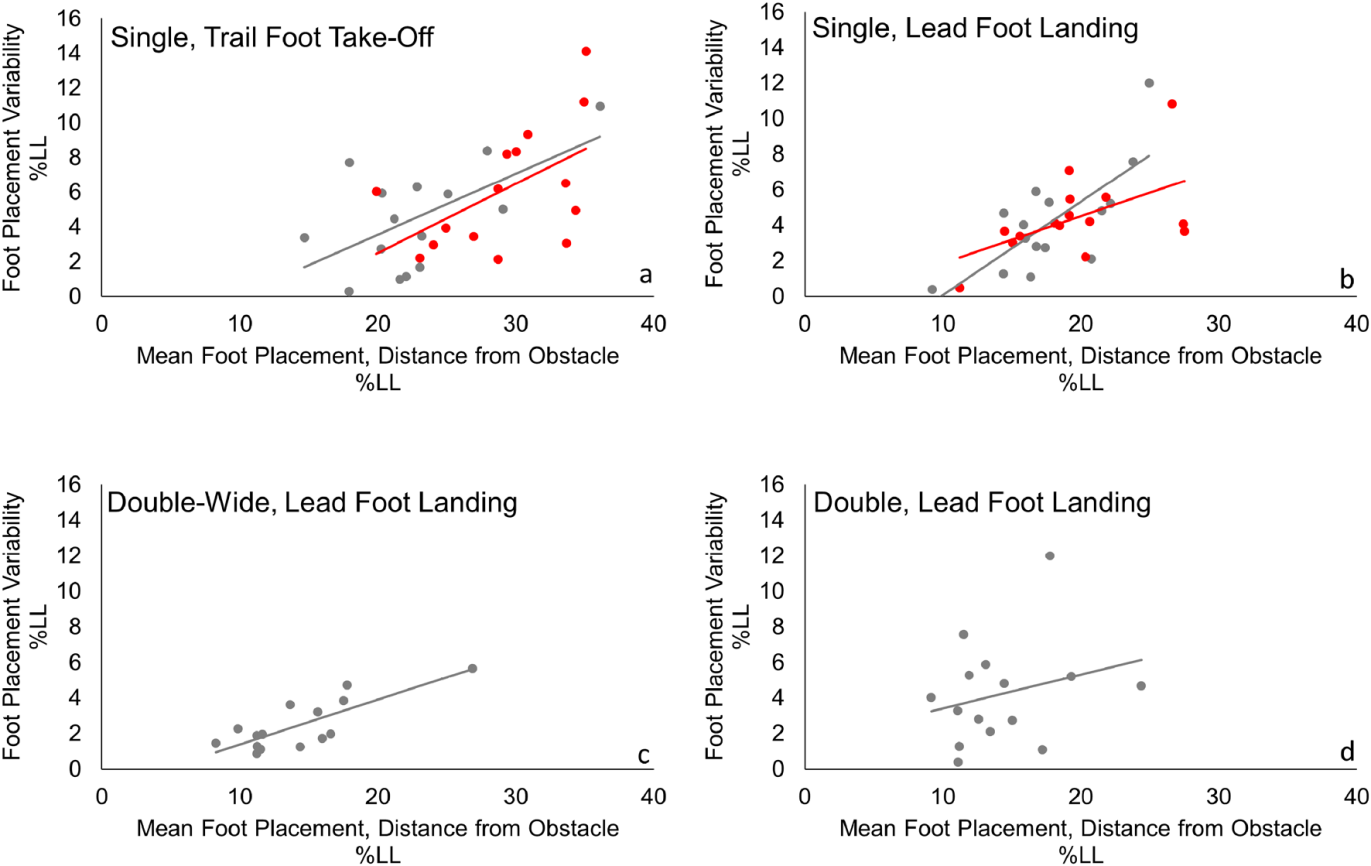
Mean distance between foot and obstacle plotted against intra-individual variability (standard deviation) for adults (black) and children (red). All values are given as a percentage of leg length (LL%)

Hypothesis 1 was partially supported*.* Children (*M* = 27.8% LL), maintained significantly greater distance between the trailing foot and the obstacle at take-off than adults (*M* = 22.5%LL); *F*(1, 28) = 10.0, *p* = 0.004, *ηp*^2^ = 0.263 (Fig. 2c). Children (*M* = 5.6% LL) also placed the trailing foot more variably at take-off than adults (*M* = 4.2% LL), *F*(1, 28) = 5.0, *p* = 0.033, *ηp*^2^ = 0.152 (Fig. 3c). Testing for dynamic scaling, we found correlations between mean take-off distance and take-off variability only in the single condition (Fig. 4a—adults, *p* = 0.018, *r* = 0.60; children, *p* = 0.04, *r* = 0.54). Hypothesis *2* was not supported*.* Neither adults nor children adjusted mean trailing foot placement in response to obstacle condition (*p* = 0.6). Obstacle condition did not affect intra-individual variability of trailing foot placement (*p* > 0.1). Obstacle condition did not interact with age for either mean or intra-individual variability of trailing foot take-off distance (*p’s* > 0.1).

**Fig. 5:**
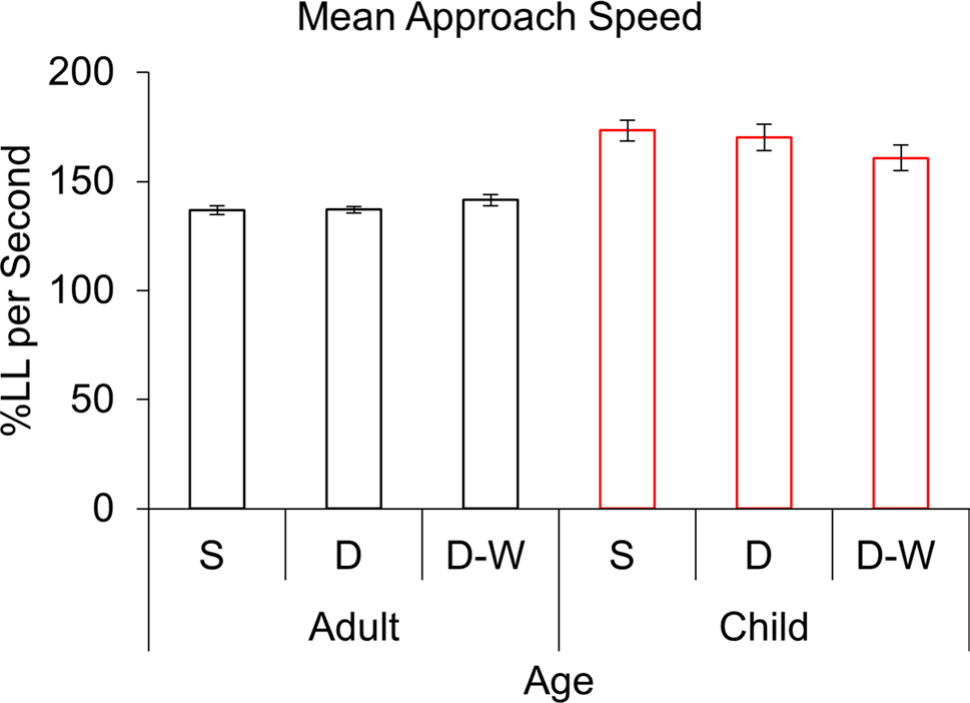
Mean approach speed presented as a percentage of leg length (LL) per second. Means are given for each condition (*S* single, *D* double, *D-W* double-wide), separately for adults (black) and children (red). Data for the leading and trailing foot are also presented separately. Error bars represent standard errors

### Approach speed

Hypothesis 2 was not supported. Mean approach speed was consistent across obstacle conditions (*p* > 0.4). However, children (*M* = 168.1% LL per second) walked significantly faster than adults (*M* = 138.5% LL per second), *F*(1, 28) = 16.3, *p* < 0.001, *ηp*^2^ = 0.368 (Fig. 5). Because of this, we introduced mean approach speed as a covariate in supplementary analyses (Supplementary Material). Importantly, statistically adjusting for speed did not result in the loss of any of our observed effects. Therefore, we conclude that our effects are not simply artefacts of differences in walking speed. There was no interaction between age and obstacle condition for mean approach speed (*p* > 0.05).

## After the first obstacle (landing)

### Leading foot

Hypothesis 1 was partially supported*.* Children (*M* = 18.8% LL) landed their leading foot significantly further forward than adults (*M* = 15.4% LL); *F*(1, 28) = 6.1, *p* = 0.02, *ηp*^2^ = 0.178 (Fig. 2b). Likewise, landing variability was higher for children (*M* = 4.9% LL) than for adults (*M* = 3.1%LL); *F*(1, 28) = 7.9, *p* = 0.009, *ηp*^2^ = 0.221 (Fig. 3b). To test for dynamic scaling, we examined correlations between means and variability. In the single obstacle condition, landing distance and landing variability for the leading foot correlated significantly for children (Fig. 4b, *p* = 0.038, *r* = 0.54) and adults (Fig. 4b, *p* = 0.002, *r* = 0.74). In the other obstacle conditions, these two measures correlated significantly for adults only (Fig. 4c, double condition: *p* = 0.005, *r* = 0.69; Fig. 4d, double-wide condition: *p* < 0.001, *r* = 0.80). Hypothesis 2 was supported*.* At both ages, obstacle condition affected leading foot placement *F*(1.6 46.143) = 7.4, *p* = 0.003, *ηp*^2^ = 0.208. The leading foot (Fig. 2b) landed further forward in the single condition (*M* = 18.8% LL) compared to the double condition (*M* = 16.1% LL), *p* = 0.011 and the double-wide condition (*M* = 16.5% LL), *p* = 0.032. Intra-individual variability of leading foot placement at landing was consistent across obstacle conditions (*p* > 0.2). Age and obstacle condition did not have an interacting effect on mean landing placement for the leading foot (*p* > 0.1). However, they did interact for intra-individual variability on this measure *F*(2, 56) = 3.7, *p* = 0.031, *ηp*^2^ = 0.116 (Fig. 3b). Specifically, children’s leading foot placement was more variable at landing in the double (*M* = 4.3% LL) and double-wide (*M* = 5.9% LL) conditions than adults’ (double *M* = 2.6% LL, *p* = 0.03; double-wide *M* = 2.5%LL, *p* = 0.003).

### Trailing foot

Hypothesis 1 was not supported*.* There was no effect of age on mean trailing foot placement at landing (*p* > 0.9), or on intra-individual variability of this measure (*p* = 0.051). Hypothesis 2 was supported*.* There was a significant main effect of obstacle condition on trailing foot placement at landing *F*(1.30, 36.596) = 113.6, *p* < 0.001, *ηp*^2^ = 0.802 (Fig. 2d). At both ages, the trailing foot landed significantly further forward in the single condition (*M* = 89.2% LL) than in the double condition (*M* = 59.0% LL), *p* < 0.001, or the double-wide condition (*M* = 61.0%LL), *p* < 0.001. Intra-individual variability of trailing foot placement at landing was also affected by obstacle condition *F*(1.292, 36.166) = 16.4, *p* =  < 0.001, *ηp*^2^ = 0.369 (Fig. 3d). At both ages, variability of trailing foot placement at landing was significantly greater in the single condition (*M* = 13.6% LL) than in the double condition (*M* = 6.2% LL), *p* = 0.001, or in the double-wide condition (*M* = 5.8% LL), *p* < 0.001. There was no interaction between age and obstacle condition for trailing foot mean placement or variability at landing (*p’s* > 0.2).

## Discussion

Using walking as a model system, we provide an exact analysis of children’s responses to both self-related constraints (variability) and environmental constraints (obstacles). Crucially, we shed light on this behaviour in the years bridging unsteady infant walking and more flexible, complex walking at 7 years (Berard and Vallis 2006). In partial support of Hypothesis 1, we find that 3- to 5-year-old children accommodate their high variability for safe and fluent walking, even in complex environments. However, in these complex environments, they employ broad strategies which are not uniquely tailored to their individual level of variability. In support of Hypothesis 2, we find that—like adults—children plan ahead when walking in a complex environment. Children adjust foot placement in response to visual information about upcoming environmental constraints. Walking provided a directly observable, naturalistic testbed for exploring the impact of both self-related and environmental constraints on children’s behaviour.

### Hypothesis 1

**Children will accommodate the self-related constraint of high foot placement variability**

We predicted that 3- to 5-year-old children would accommodate variable foot placement via Dynamic Scaling, even in complex, multiple obstacle environments. This prediction was partially supported. As expected, we found that children’s foot placement was significantly more variable than adults’. Further, we found that both adults and children accommodated this self-related constraint via Dynamic Scaling in single obstacle conditions (Snapp-Childs and Bingham 2009). Those with more variable foot placement maintained larger margins of error between the feet and an obstacle. This mitigates the risk of tripping on obstacles. However, in complex, multiple obstacle conditions, only adults engaged in Dynamic Scaling. In contrast, children employed large margins of error irrespective of their own individual level of variability. We argue that 3- to 5-year-old children are sensitive and responsive to the self-related constraints of high movement variability. However, in complex conditions, children’s strategy is not as energy efficient as adults’. Rather, children are variability-expectant. They use a broad, untailored strategy, producing exaggerated margins of error. Similarly, when performing multisensory tasks, young children under 8 years show broad reliance on a single most reliable sense rather than tailoring their reliance on multiple senses according to the relative variability of each sensory modality (Gori et al. 2008; Nardini et al. 2008).

Variability is a self-related constraint to be controlled, especially for safe movement through complex environments. However, there are also inherent benefits of variability for children’s learning (Gliga 2018). New walkers engage in frequent bouts of walking of variable path and duration: the variability is to the benefit of motor learning (Adolph et al. 2012). Likewise, infants spontaneously banging objects on a surface do so with greater variability when given a more complex object to hold (Kahrs and Lockman 2014). Under conditions of complexity, variability may be particularly beneficial for exploring new possibilities and identifying the most efficient or useful patterns of action. In the present study, we observed more variable foot placement among 3- to 5-year-old children than adults. This was especially true in the more complex, double obstacle conditions. High variability allows young children to explore numerous possible foot placement strategies for crossing multiple obstacles. With time, the most appropriate strategies can be selected and fine-tuned. At 3- to 5-years of age, children have a little way still to go. With practice, they must refine their behaviour so that it can become more efficient.

### Hypothesis 2

**Children will plan ahead-even in complex environments**

We predicted that children would plan ahead in a complex environment. In our task, this would be shown by adjustments of foot placement around the first obstacle depending on the upcoming obstacle sequence (Berard and Vallis 2006; Krell and Patla 2002; Matthis et al. 2017), and by reducing walking speed ahead of the obstacle. Our hypothesis was supported. At 3- to 5-years, children made adjustments in response to proximal environmental constraints. For example, children brought the trailing foot tightly over the first obstacle when a second obstacle was to follow. In further support of the hypothesis, children planned for multiple constraints which were more distant (spatially and temporally). When approaching the first obstacle – and a full four steps in advance of the second obstacle—both children and adults placed the leading foot more carefully (with lower variability) when there was more complex terrain ahead. We also expected children to reduce walking speed when approaching obstacles. However, we did not find statistical support for this.

Our work builds on previous research with both older and younger children. Previous work has elegantly demonstrated that toddlers make developmentally-appropriate choices when directly faced with environmental constraints such as slopes, barriers and unusual surfaces (Adolph et al. 1993; Gibson et al. 1987; Schmuckler 1996). In these cases, the relevant visual information is simple (single obstacle) and immediately available. In a more complex, multiple-obstacle environment, we find that children, who are only a little older, make adaptive motor plans. These plans are made well in advance, in response to distal visual information about multiple, upcoming constraints. This appears to contrast with previous work showing that older 7-year-old children plan for crossing a second obstacle only after crossing the first (Berard and Vallis 2006). However, this work (Berard and Vallis 2006) used obstacles and configurations which were not scaled to leg length. Therefore, the obstacles and distances between obstacles were proportionately larger than those used for children in the present study. This larger inter-obstacle distance may have allowed children to make additional adjustments in between obstacles (Berard and Vallis 2006) which were not possible in the present study.

We have shown that 3- to 5-year-old children plan ahead during walking. Crucially, they do so on the basis of distal visual input about the environment. Our findings complement limited eye-tracking evidence that 4- to 8-year-olds (and adults) visually sample objects from around 3 steps ahead when freely navigating cluttered environments (Franchak and Adolph 2010). In a complex, multi-obstacle environment, children generally plan for obstacles in very similar ways to adults. This builds upon our understanding of early motor planning. Whilst infants show sophisticated prospective control of seated reaching movements (Clifton et al. 1991; Gottwald et al. 2017; Gottwald and Gredebäck 2015), by the pre-school age, children are capable of planning multi-step actions in complex, multiple-obstacle environments in the unstable upright posture of walking.

## Conclusions

Using walking as a testbed, we detail the strategies that 3 to 5-year-olds use to accommodate both self-related constraints (variability) and environmental constraints (obstacles) to produce skilled, flexible behaviour. Children were sensitive to their variable foot placement (Hypothesis 1). They accommodated this with large margins of error between their feet and the obstacle. However, as per other multisensory tasks, children did not always tailor their responses to their unique levels of variability (Gori et al. 2008; Nardini et al. 2008). Like adults, children planned ahead during walking (Hypothesis 2). They adjusted the position and the variability of foot placement well in advance of upcoming obstacles. This illustrates that, despite the complexity of human bipedal walking, young children learn to control their locomotion in a skilled and efficient manner. They optimise their ability to cope with varied and complex everyday environments.

## Availability of data, code and materials

Data will be made openly available on the Durham University data repository at: 10.15128/r13t945q809

## Electronic supplementary material

Below is the link to the electronic supplementary material.

Supplementary file1 (DOCX 16 kb)

## References

[CR1] Adalbjornsson CF, Fischman MG, Rudisill ME (2008). The end-state comfort effect in young children. Res Q Exerc Sport.

[CR2] Adolph KE, Eppler MA, Gibson EJ (1993). Crawling versus walking infants’ perception of affordances for locomotion over sloping surfaces. Child Dev.

[CR3] Adolph KE, Vereijken B, Shrout PE (2003). What changes in infant walking and why what changes in infant walking and why two of the central goals of developmental. Sour Child Dev Child Dev.

[CR4] Adolph KE, Cole WG, Komati M, Garciaguirre JS, Badaly D, Lingeman JM, Sotsky RB (2012). How do you learn to walk? Thousands of steps and dozens of falls per day. Psychol Sci.

[CR5] Adolph KE, Hoch JE, Cole WG (2018). Development (of walking): 15 suggestions. Trends Cogn Sci.

[CR6] Berard JR, Vallis LA (2006). Characteristics of single and double obstacle avoidance strategies: a comparison between adults and children. Exp Brain Res.

[CR7] Berger SE, Adolph KE (2003). Infants use handrails as tools in a locomotor task. Dev Psychol.

[CR8] Chen HC, Ashton-Miller J, Alexander N, Schultz A (1994). Age effects on strategies used to avoid obstacles. Gait Posture.

[CR9] Clifton RK, Rochat P, Litovsky RY, Perris EE (1991). Object representation guides infants' reaching in the dark. J Exp Psychol Hum Percept Perform.

[CR10] Cole WG, Robinson SR, Adolph KE (2016). Bouts of steps: the organization of infant exploration. Dev Psychobiol.

[CR11] Corporaal SHA, Swinnen SP, Duysens J, Bruijn SM (2016). Slow maturation of planning in obstacle avoidance in humans. J Neurophysiol.

[CR12] Field A (2013) Discovering statistics using IBM SPSS statistics. SAGE Publications, London

[CR13] Franchak JM, Adolph KE (2010). Visually guided navigation: head-mounted eye-tracking of natural locomotion in children and adults. Vision Res.

[CR14] Franchak JM, Kretch KS, Soska KC, Adolph KE (2011). Head-mounted eye tracking: a new method to describe infant looking. Sour Child Dev Child Dev.

[CR15] Gibson EJ, Riccio G, Schmuckler MA, Stoffregen TA, Rosenberg D, Taormina J (1987). Detection of the traversability of surfaces by crawling and walking infants. J Exp Psychol Hum Percept Perform.

[CR16] Gliga T (2018) Telling apart motor noise from exploratory behaviour, in early development. Front Psychol 9:1939 10.3389/fpsyg.2018.01939 PMC6194153 30369897

[CR17] Godoi D, Barela J (2008). Body sway and sensory motor coupling adaptation in children: effects of distance manipulation. Dev Psychobiol.

[CR18] Gori M, Del Viva M, Sandini G, Burr DC (2008). Young children do not integrate visual and haptic form information. Curr Biol.

[CR19] Gottwald JM, Gredebäck G (2015). Infants’ prospective control during object manipulation in an uncertain environment. Exp Brain Res.

[CR20] Gottwald JM, De Bortoli VA, Lindskog M, Nyström P, Ekberg TL, von Hofsten C, Gredebäck G (2017). Infants prospectively control reaching based on the difficulty of future actions: To what extent can infants’ multiple-step actions be explained by Fitts’ law?. Dev Psychol.

[CR21] Hausdorff JM, Zemany L, Peng C, Goldberger AL (1999). Maturation of gait dynamics: stride-to-stride variability and its temporal organization in children. J Appl Physiol.

[CR22] Von Hofsten C (2007). Action in development. Dev Sci.

[CR23] Ivanenko YP, Dominici N, Cappellini G, Dan B, Cheron G, Lacquaniti F (2004). Development of pendulum mechanism and kinematic coordination from the first unsupported steps in toddlers. J Exp Biol.

[CR24] Kahrs BA, Lockman JJ (2014). Building tool use from object manipulation: a perception-action perspective. Ecol Psychol Publ Int Soc Ecol Psychol.

[CR25] Krell J, Patla AE (2002). The influence of multiple obstacles in the travel path on avoidance strategy. Gait Posture.

[CR26] Lee DK, Cole WG, Golenia L, Adolph KE (2018). The cost of simplifying complex developmental phenomena: a new perspective on learning to walk. Dev Sci.

[CR27] Matthis JS, Barton SL, Fajen BR (2017). The critical control phase for the visual control of walking over complex terrain. PNAS.

[CR28] Matthis JS, Yates JL, Hayhoe MM (2018). Gaze and the control of foot placement when walking in natural terrain. Curr Biol.

[CR29] Nardini M, Jones P, Bedford R, Braddick O (2008). Development of cue integration in human navigation. Curr Biol.

[CR30] Nardini M, Begus K, Mareschal D (2013). Multisensory uncertainty reduction for hand localization in children and adults. J Exp Psychol Hum Percep Perform.

[CR31] Okamoto T, Okamoto K, Andrew PD (2003). Electromyographic developmental changes in one individual from newborn stepping to mature walking. Gait Posture.

[CR32] Patla AE, Vickers JN (1997). Where and when do we look as we approach and step over an obstacle in the travel path?. NeuroRep.

[CR33] Patla AE, Vickers JN (2003). How far ahead do we look when required to step on specific locations in the travel path during locomotion?. Exp Brain Res.

[CR34] Patla AE, Prentice SD, Robinson C, Neufeld J (1991). Visual control of locomotion: strategies for changing direction and for going over obstacles. J Exp Psychol Hum Percept Perform.

[CR35] Rosenbaum D, Chapman K, Weigelt M, Weiss D, Van der Wel R (2013). Cognition, action and object manipulation. Psychol Bull.

[CR36] Schmuckler MA (1996). Development of visually guided locomotion: barrier crossing by toddlers. Ecol Psychol.

[CR37] Snapp-Childs W, Bingham GP (2009). The affordance of barrier crossing in young children exhibits dynamic, not geometric, similarity. Exp Brain Res.

[CR38] Sutherland D (1997). The development of mature gait. Gait Posture.

[CR39] Woollacott MH, Shumway-Cook A (1990). Changes in posture control across the life span—a systems approach. Phys Ther.

[CR40] Zelazo P, Carter AS (1997). Early development of executive function: a problem-solving framework child and family development project view project visual search in toddlers with ASD View project. Art Rev GenPsychol.

