# Supplementary material for: Mind your step: learning to walk in complex environments
Source: Exp Brain Res. 2020 May 13;238(6):1455–65. doi: 10.1007/s00221-020-05821-y (PMC7286854; doi:10.1007/s00221-020-05821-y)
Supplement: Supplementary file 1 — Supplementary file1 (DOCX 16 kb) [file 221_2020_5821_MOESM1_ESM.docx]

**Supplementary Material**

The data was adjusted using the covariate Mean Approach Speed. For each participant, each dependent variable was averaged across the 3 conditions (Single, Double, Double-wide). This left each participant with 8 ‘scores’ (one per dependent variable- *1 Leading foot mean take-off distance, 2 Trailing foot mean take-off distance, 3 Leading foot mean landing distance, 4 Trailing foot mean landing distance, 5 Leading foot take-off distance variability, 6 Trailing foot take-off distance variability, 7 Leading foot landing distance variability, 8 Trailing foot landing distance variability*). Each of these new scores were regressed against Mean Approach Speed (as a %LL per second). The resulting unstandardized residuals for each variable were added to the raw data for that variable- this adjusted the data for speed i.e. removed any variability within the data which could be attributed to speed. Importantly, this process ensures that the relationships between the different conditions remain unchanged. The now-adjusted data was used to conduct a series of mixed model ANOVAs as per the original analysis. Effects of condition and interactions are unchanged from analyses in the main text. Some effects of age - which were not significant in the original analysis - were significant following the adjustment for speed. These are summarised in Table 1 below.

**Table 1** Age effects before and after adjusting for speed

| **Variable** | **Unadjusted**  **Age Effect** | **Adjusted**  **Age Effect** |
| --- | --- | --- |
| Leading Foot - Mean Take-Off Distance | *p*=.225 | *F*(1, 28)=18.153, *p*<.001, η*^2^*=0.393 |
| Leading Foot – Take Off Distance Variability | *p*=.086 | *F*(1, 28)=24.085, *p*<.001, η^2^=0.462 |
| Trailing Foot – Mean Landing Distance | *p*=.938 | *F*(1, 28)=13.083, *p*<.001, η^2^=0.318 |
| Trailing Foot – Landing Distance Variability | *p*=.051 | *F*(1, 28)=19.876, *p*<.001, η^2^=0.415 |
